# The Impact and Consequences of SARS-CoV-2 Pandemic on a Single University Dermatology Outpatient Clinic in Germany

**DOI:** 10.3390/ijerph17176182

**Published:** 2020-08-26

**Authors:** Rosi Wang, Charlotte Helf, Linda Tizek, Ruth Neuhauser, Kilian Eyerich, Alexander Zink, Bernadette Eberlein, Tilo Biedermann, Knut Brockow, Alexander Boehner

**Affiliations:** 1 Department of Dermatology and Allergy Biederstein, Faculty of Medicine, Technical University Munich, 80802 Munich, Germany; rosi.wang@tum.de (R.W.); charlotte.helf@uni-rostock.de (C.H.); linda.tizek@tum.de (L.T.); ruth.neuhauser@tum.de (R.N.); kilian.eyerich@tum.de (K.E.); alexander.zink@tum.de (A.Z.); bernadette.eberlein@tum.de (B.E.); tilo.biedermann@tum.de (T.B.); knut.brockow@tum.de (K.B.); 2 Division of Dermatology and Venereology, Department of Medicine Solna, and Center for Molecular Medicine, Karolinska Institutet, 171 77 Stockholm, Sweden; 3 Unit of Dermatology, Karolinska University Hospital, 171 64 Solna, Sweden

**Keywords:** outpatient care, COVID-19 (coronavirus disease 2019), SARS-CoV-2 (severe acute respiratory syndrome coronavirus 2), public health

## Abstract

The pandemic outbreak of coronavirus disease 2019 (COVID-19) affects health care systems globally and leads to other challenges besides infection and its direct medical consequences. The aim of this study was to investigate the impact of SARS-CoV-2 (severe acute respiratory syndrome coronavirus 2) pandemic on the university dermatology outpatient clinic (UDOC) of the Technical University of Munich, Germany. We analyzed datasets from 2015 until 2020 extracted from the hospital information system database and our documented outpatient files regarding patient numbers, gender, age, and diagnoses. In 2020, case numbers of outpatient care declined significantly (*p* = 0.021) compared to previous years and was related to the timing of political announcements answering SARS-CoV-2 pandemic. Additionally, during calendar week 10 to 15—the peak time of the spread of COVID-19 in Germany—the proportion of patients missing their consultation was significantly higher in 2020 than in 2019 (22.4% vs. 12.4%; *p* < 0.001). Gender-associated differences regarding absences were not detected, but patients aged 85 years or older were significantly more likely to miss their consultation compared to all other age groups (*p* = 0.002). Regarding different disease clusters, patients with chronic inflammatory skin diseases and infectious and malignant diseases were more likely to miss their consultation (*p* = 0.006). Noticeably, less patients with malignant diseases, and particularly malignant melanoma, were registered during this pandemic. Our data support the hypothesis that medically constructive prioritization might not be implemented properly by patients themselves. Identifying missed patients and catching up on their medical care apart from COVID-19 will pose an enormous challenge for health care systems globally.

## 1. Introduction

Coronavirus disease 2019 (COVID-19) is an infectious disease caused by severe acute respiratory syndrome coronavirus 2 (SARS-CoV-2), which first occurred in December 2019 [1,2].

The pandemic spread of SARS-CoV-2 definitely shapes not only worldwide public life, politics and economics, it also strongly affects health care systems globally and leads to other challenges besides infection and its direct medical consequences. Limited medical resources oblige politicians and the general public to take rigorous measures quickly [3].

While the primary focus was on providing best medical care to critically ill COVID-19 patients by ensuring sufficient capacities of intensive care unit beds and on enacting regulations to embank the pandemic, regular outpatient care in all other medical specialties was affected adversely. Moreover, close and extensive reporting by the media was ubiquitous: On March 16, 2020, a state of emergency was declared in the state Bavaria, Germany, with introduction of both exit and contact restrictions by the government [4].

In order to determine the impact of SARS-CoV-2 pandemic on dermatological patients, this study aimed to investigate the changes of case and patient numbers in the university dermatology outpatient clinic (UDOC) of the Technical University of Munich, Germany, during the pandemic compared to previous years.

## 2. Methods

This monocentric, non-interventional, retrospective study was approved by the ethics committee of the Faculty of Medicine at the Technical University of Munich (reference 270/20).

This study was conducted according to a retrospective study design that utilized datasets extracted from the hospital information system database and documented outpatient files. First, all patient cases seen in the UDOC between January and April from 2015 to 2020 were analyzed by extracting case numbers and the corresponding International Classification of Diseases (ICD) codes. To determine the time-dependent impact of SARS-CoV-2 pandemic on patient care, case numbers per week were analyzed between January (beginning from calendar week 2 (CW 2)) and April (until CW 16) from 2015 to 2020 (15 weeks in total). The UDOC consists of five different subunits including dermato-oncology, allergology, special consultation-hours for example in autoimmune clinics, phototherapy, and the unselected dermatologic outpatient clinic, referred to as polyclinic (PC). Thereby, the PC functions as a kind of triage, where especially initial but also consecutive consultations take place. After the initial consultation patients are distributed to one of the other subunits whenever needed. As a result, the PC constitutes the major share of the UDOC and was analyzed more detailed in the following.

As multiple visits of an individual patient within the same quarter (three months) are summarized to one case due to accounting reasons, real numbers of consultations are higher than case numbers coded. Consequently, real numbers of consultations between March and April 2019 and 2020 (CW 10 until CW 15) were determined by working through the time schedule of the polyclinic (PC). Moreover, diagnoses were specified by reviewing documented records in the outpatient files, which raised the quality of the data.

Real numbers of actual consultations were evaluated subsequently and collated with those missed in the above-named time range in 2020 compared to 2019. Furthermore, distribution of sex, age, and different diagnose clusters in the patient collective were investigated; patients were divided into patients who attended their consultation and those who missed their consultation (referred to as “no-shows” in the following), respectively. Clusters of diagnoses included malignant, autoimmune, inflammatory, allergic/reactive, infectious diseases, benign neoplasia, various, and not classifiable diseases. Not classifiable diagnoses resulted from those patients where a clear diagnosis could not be made from the data available or patients missing their initial consultation.

### Statistical Analyses

Statistical analyses were performed using GraphPad Prism in the Version of 8.4.2 (GraphPad Software Inc., San Diego, CA, USA) and IBM SPSS version 25 (IBM Corporation, Armonk, NY, USA). To assess differences in no-shows and incoming patients in 2020, categorical variables were compared using a chi-squared test and continuous variables using a *t*-test. Furthermore, a one-way analysis of variance (ANOVA) with a Bonferroni post-hoc test was applied to examine differences in number of patients within the years. Significance level was defined as *p* < 0.05 (*), *p* < 0.01 (**), and *p* < 0.001 (***).

## 3. Results

### 3.1. Number of Outpatient Cases Declined Significantly as a Consequence of the SARS-CoV-2 Pandemic

Overall, a total of 49,161 cases presented to the UDOC between January and April from 2015 to 2020, cumulatively. While there was an average of 8471 cases recorded between CW 2 and CW 16 in the years from 2015 to 2019, only 6805 cases were recorded in the same time range in 2020 (Figure S1A,B). Interestingly, the distribution of the different subunits within the UDOC did not change (Figure S1C). Since the unselected dermatologic outpatient clinic, referred as polyclinic (PC), does not only constitute the major share of the UDOC but also represents the “common” dermatology patients with their various diagnoses in the most appropriate way, it was analyzed more detailed in the following.

**Figure 1:**
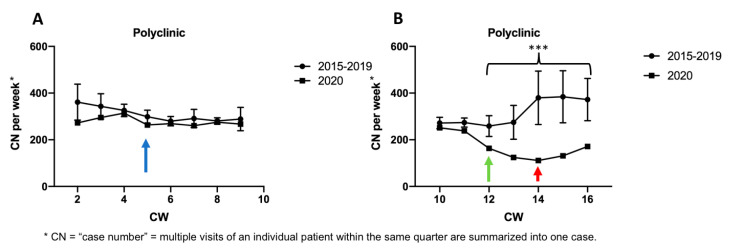
CN per week of the polyclinic. (**A**) Average CN per week between CW 2 and CW 9 from 2015 to 2019 compared to 2020. (**B**) Average CN per week between CW 10 and CW 16 from 2015 to 2019 compared to 2020. First case of COVID-19 reported in Bavaria in CW 5 (blue arrow), declaration of state of emergency in Bavaria in CW 12 (green arrow), beginning of the second accounting quarter in CW 14 (red arrow). Significance level was defined as *p* < 0.001 (***). CN, case number; CW, calendar week.

Between CW 2 to CW 16 in 2020, significantly less patients were treated in the PC (*p* < 0.001) compared to the previous years (3404 cases in 2020 vs. 4685 cases on average from 2015 to 2019), reflecting a decline of almost 30% (Figure 1).

Slightly declining patient cases were observed in 2015–2019 between CW 2 and CW 13 in the PC (Figure 1) with a substantial increase of case numbers at the beginning of the second accounting quarter in CW 14 (Figure 1A, red arrow). In 2020 rather constant case numbers during CW 2 to CW 11 with a minor drop in CW 5 (Figure 1A, blue arrow) were observed, when the first case of COVID-19 in Bavaria occurred on 27 January 2020. Interestingly, a clear temporal coincidence was apparent between reduced patient attendance and political announcements (Figure 1B, green arrow) with a significant reduction in case numbers between CW 12 to CW 16 (*p* < 0.001) in 2020: On March 16th (beginning of CW 12) a state of emergency was declared in Bavaria. Additionally, the expected increase in case numbers in CW 14 at the beginning of the second accounting quarter observed during the previous years was absent in 2020 (Figure 1B, red arrow).

### 3.2. Substantial Increase of Missed Consultations during the Peak of SARS-CoV-2 Pandemic

**Figure 2:**
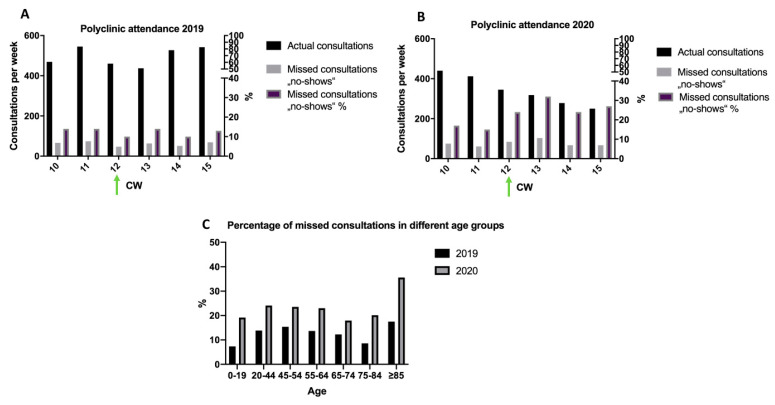
Polyclinic attendance. (**A**) Consultations per week between CW 10 and CW 15 in 2019. Actual consultations per week (black column) and missed consultations (no-shows; gray column) in the polyclinic presented on the left y-axis. Relative proportion of no-shows compared to total number of consultations (violet column) in the polyclinic illustrated on the right y-axis. Enforcement of exit and contact restrictions in Bavaria in CW 12 (green arrow). (**B**) Consultations per week between CW 10 and CW 15 in 2020. Actual consultations per week (black column) and missed consultations (no-shows; gray column) in the polyclinic presented on the left y-axis. Relative proportion of no-shows compared to total number of consultations (violet column) in the polyclinic illustrated on the right y-axis. Enforcement of exit and contact restrictions in Bavaria in CW 12 (green arrow). (**C**): Percentage of missed consultations (no-shows) in the polyclinic clustered in different age groups between March and April 2019 (black) and 2020 (gray). CW, calendar week.

While average number of actual consultations per week during CW 10 and CW 15 within the PC were rather consistent in 2019, a progressing decline was observed in 2020. During CW 10 to CW 15, the proportion of patients missing their consultation (“no-shows”) was significantly higher in 2020 (22.4% of 2043 total consultations) compared to 2019 (12.4% of 2979 total consultations; *p* < 0.001; Figure 2A,B). The highest share of no-shows in 2020 was observed in relation to a temporal connection to exit and contact restrictions, which came into force on 20 March 2020in CW 12, with nearly every third consultation being missed in the following CW 13 (Figure 2B).

### 3.3. Patient Groups Were Differently Influenced by SARS-CoV-2 Pandemic

The distribution of demographical variables of PC patients was investigated during the peak months of the pandemic from March to April 2020 compared to the equivalent time range in 2019 by analyzing actual consultations and missed consultations, respectively.

In general, slightly more male patients were seen in the PC with 55.17% (Figure S2C) in 2020 compared to 49.75% in 2019 (Figure S2A), respectively, being male. Interestingly, no significant differences in characteristics of no-shows were observed regarding gender (63.8% male in 2019 (Figure S2B) vs. 59.3% in 2020 (Figure S2D), *p* = 0.188).

Regarding the average age in March and April 2019 (Figure S3A) compared to 2020 (Figure S3B), there were no significant changes detected neither in the group of the attending patients (46.26 ± 23.58 years in 2019 vs. 47.06 ± 23.08 years in 2020) nor in the group of the no-shows (47.04 ± 20.66 years in 2019 vs. 46.60 ± 23.19 years in 2020).

However, clustering of patients into different age groups revealed higher no-show rates in 2020 than in 2019 with people aged > 85 years being most likely to miss the consultation. Belonging to the oldest age group in 2020 was significantly associated with missing the consultation compared to all other age groups (*p* = 0.001; Figure 2C).

A relative shift towards malignant, chronic inflammatory, and infectious skin diseases was detected in the group of no-shows in 2020.

**Figure 3:**
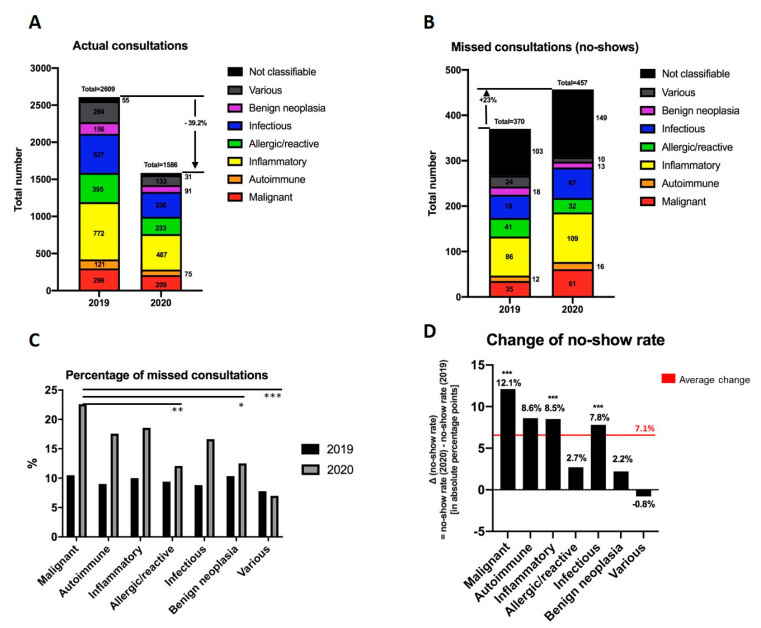
Distribution of diagnosis clusters in the polyclinic in March and April 2019 and 2020 within the group of actual and missed consultations. (**A**,**B**) Absolute numbers: (**A**) Actual consultations; (**B**) missed consultations (no-shows), consisting of malignant (red), autoimmune (orange), inflammatory (yellow), allergic/reactive (green), infectious (blue), various (gray), not classifiable (black) diseases, and benign neoplasia (violet). (**C**,**D**) Relative proportion of no-shows to total consultations: (**C**) Percentage of missed consultations in 2019 (black) and 2020 (gray); (**D**) change of no-show rate 2020 compared to 2019 indicated in absolute percentage points. Average change indicated in red. Significance level was defined as *p* < 0.05 (*), *p* < 0.01 (**), and *p* < 0.001 (***).

Consistent with the declining case numbers in the PC (Figure 1), a decrease of 39.2% of actual consultations was noted during the peak time of the pandemic in March and April (1586 total consultations in 2020 vs. 2069 consultations in 2019; Figure 3A). Thereby, actual consultations decreased considerably in each and every diagnosis category. In addition, the number of missed consultations increased by 23% from 370 in 2019 to 457 in 2020 (Figure 3B).

In order to detect if patients with certain diagnoses might be more likely to miss their consultation, the distribution of diagnosis clusters in the group of no-shows in particular was analyzed. In 2019, 28% (*n* = 103) and in 2020 33% (*n* = 149) of the no-shows were initial consultations where no accurate diagnoses could be made due to absence of the patient and therefore were categorized as “not classifiable.” Not classifiable diagnoses were excluded in further analyses.

In 2019, there was no significant difference within the diagnosis groups regarding the no-show rate (*p* = 0.902; Figure 3C). However, within the year 2020 a significant difference regarding the no-show rate was detected with patients suffering from a malignant diagnoses being more likely to miss their consultation compared to patients with an allergic diagnosis (*p* = 0.001) and to consultations regarding benign neoplasia (*p* = 0.028; Figure 3C).

Comparing no-show rates of different diagnosis groups between the years 2020 and 2019 revealed the highest increase in the group of malignant diseases. While an average increase of 7.1 absolute percentage points was detected in all entities in 2020 compared to 2019, an increase of 12.1 absolute percentage points was detected in the group of malignant diseases (Figure 3D). When comparing the different diagnosis clusters in 2020 to 2019, no-show rates increased significantly in the group of malignant (*p* < 0.001), inflammatory (*p* < 0.001), and infectious (*p* < 0.001) diseases (Figure 3D).

### 3.4. Absolute Case Numbers of Certain Diagnoses in Our PC Decreased during SARS-CoV-2 Pandemic

**Figure 4:**
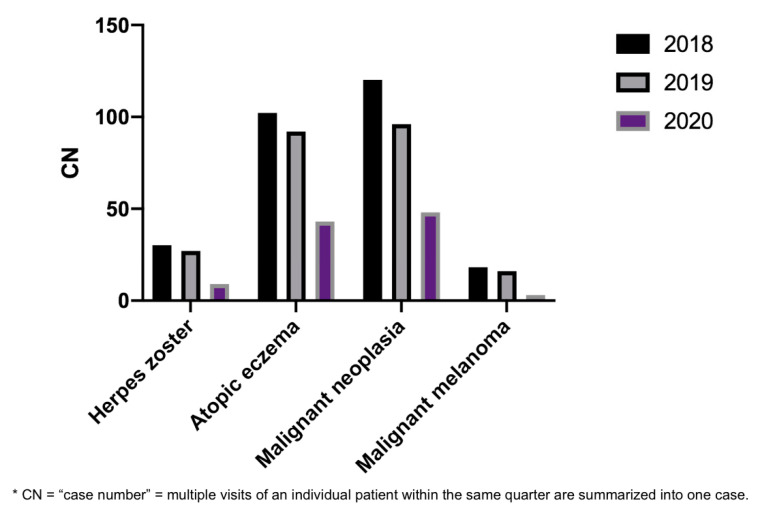
Absolute CN of herpes zoster, atopic eczema, malignant neoplasia, and malignant melanoma in March and April in 2018 (black column), 2019 (gray column), and 2020 (violet column). CN, case number.

Absolute numbers of representative acute (herpes zoster), chronic (atopic eczema), and malignant (malignant neoplasia) diagnoses in the PC were lower in March and April 2020 compared to 2019 and 2018, respectively (Figure 4). While an average of 29 cases of herpes zoster were seen in 2018 and 2019, less than half of those cases were registered in 2020 (*n* = 9). The same applies to atopic eczema; compared to the average of 97 cases in 2018 and 2019, a decline to 54 cases was observed in 2020.

With 48 cases of malignant neoplasia in 2020, less than half of the diagnoses were documented compared to the two previous years. By comparing the number of patient cases of malignant melanoma from 2020 to the previous two years, a reduction of 82.35% was detected as only three cases of malignant melanomas were seen in the investigated time range of 2020. In contrast, 18 cases were recorded in 2018 and 16 in 2019 within the same period in the PC (Figure 4).

## 4. Discussion

These results demonstrated a significant drop of cases in the UDOC in March and April 2020 compared to previous years, which were time related to the SARS-CoV-2 pandemic and the regulations enacted. While a substantial increase of missed consultations during the peak of SARS-CoV-2 pandemic in general was revealed, various risk groups were differently influenced. Unlike age, gender was not detected as a variable affected by the pandemic in terms of the probability of missing a consultation. In contrast, patients aged 85 years or older notably missed their consultation significantly more often. In addition, especially patients with malignant, inflammatory and infectious diseases missed their consultation more frequently in 2020. Most importantly, absolute case numbers of certain diagnoses, e.g., malignant melanoma, decreased substantially during SARS-CoV-2 pandemic.

Regular fluctuations of patient attendance in different outpatient clinics have already been observed before the worldwide spread of COVID-19. This variability can be attributed to general parameters such as public holidays and weekdays, weather, or staffing [5,6,7,8]. However, the usual variability in case numbers does not explain the striking difference observed in March and April 2020. Previous studies have already highlighted the impacts of SARS-CoV-2 pandemic on medical consultations and admissions. For instance, admissions for acute coronary syndrome declined significantly in Austria since its outbreak [9,10]. Furthermore, a recent study reported on a significantly reduced patient volume of 30% in an emergency department [11]. Consistent with those results, this study shows an obvious drop of cases seen in the UDOC of the Technical University in Munich, Germany, in March and April 2020 compared to previous years. This drop was directly time related to political announcements; following 16 March 2020, when a state of emergency was declared by the minister of Bavaria [4], case numbers decreased significantly compared to the average case numbers from 2015 until 2019. However, general outpatient care might have been not only unintentionally influenced by strict public health regulations, which undoubtedly are crucial points in controlling the spread of COVID-19, but also by extensive media coverage regarding infection numbers and including pictures of hospitals with overburdened health care workers and risks for visiting or admitted patients during the peak time of this pandemic [12].

The observed phenomenon of continuously decreasing case numbers in 2015 to 2019 during the first quarter with a pronounced rise at the beginning of the second quarter can be attributed to the German health system accounting modality. In Germany, one patient case is deducted following the first consultation of a patient in each quarter. Any subsequent consultation in the same quarter will not be represented in the case numbers anymore. Thus, decreasing case numbers do not automatically reflect declining consultations in the clinic, which is supported by more precise analyzes of actual patient numbers and therefore “actual consultations”.

Therefore, real patient numbers in the PC were investigated by processing and analyzing actual and missed consultations, respectively. These more detailed, and time-dependent analyses revealed a progressing decrease of actual consultations and a relative increase of missed consultations after the enactment of exit and contact restrictions. This might be due to the fact that patients were more reserved to schedule and attend consultations, during this peak phase of the pandemic.

As male gender and older age were communicated to be risk factors associated with hospitalization and death [13,14,15], one could hypothesize that male and older patients were more likely to miss their consultations. Surprisingly, in this study gender-related shifts were not seen. However, higher age was identified as a variable affecting the likelihood to miss medical consultations.

Interestingly, regarding diagnosis clusters, no clear shift in favor towards those diseases which are generally considered more urgent and threatening, such as malignant diseases, was observed. In contrast, no-show rates increased significantly in patients with malignant, inflammatory, and infectious diseases. This increase was most pronounced in the group of malignant diseases with nearly every forth consultation being missed in 2020. Discussions about cancer as a possible risk factor for severe outcome of COVID-19 infection might have unsettled cancer patients and prevented them from attending their consultation [16,17]. Thus, these results indicate, that medically constructive prioritization might not be implemented properly by patients themselves when deciding if a medical consultation is necessary or not [10].

Most importantly, a pronounced decline regarding absolute numbers of certain diagnoses was observed during the peak phase of the pandemic. Evaluation of representative acute, chronic, and malignant skin diseases revealed a decrease in number of cases in each and every entity investigated. While postponed treatments or diagnoses of e.g., atopic eczema as a chronic disease do not have very far-reaching consequences, certainly, not properly treated herpes zoster may entail the risk of postzoster neuralgia and cardiovascular events [18,19]. Moreover, concerns should be raised in terms of the alarming decline of malignant diseases and in particular malignant melanoma. While 18 cases with malignant melanoma were documented in 2018 and 16 in 2019, only three cases with malignant melanoma were seen in 2020 within the same period in March and April. Since early malignant melanoma is an asymptomatic disease, patients’ awareness regarding the medical necessity of a consultation might be underestimated [20]. However, especially in regard to malignant diseases, it is of utmost importance that diagnoses are made at the earliest stage possible and that therapeutic treatment interruptions, which could possibly affect medical outcomes, are avoided [21]. A recent study predicted a substantial increase in the number of avoidable cancer cases in England as a result of diagnostic delays already by now due to the pandemic [22].

Interestingly, a modest increase of cases from CW 15 in 2020 was observed, which might be a reaction to decreasing COVID-19 infection numbers at that time in Germany, but also a sign of patients getting used to the pandemic and starting to live in the new normality. Apart from that, this observation might also partly result from catch-up effects; some of those patients who presented at that time might have been forced to see a doctor because of the aggravation of their disease, which did not allow them to postpone their consultation any longer.

Our focus on solely dermatology patients, allowed us to specify and categorize individual diagnoses. Therefore, more precise analyses were performed, and we did not rely on coded data only.

Certainly, this study contains limitations. First, it is a monocentric study. With this, local influences like weather, holidays and weekdays, or transportation disruption cannot be excluded. However, a strong impact on the outcome is unlikely. Second, data was obtained merely retrospectively from the hospital information system database using secondary coding data and outpatient files, respectively. This narrows the verifiability as neither doctors nor patients were interviewed. However, fewer consultations and therefore diagnosed and treated patients are alarming. Third, various actions to impede the spread of COVID-19 within the clinic were taken, which might have also influenced patient attendance. An automatic reply to e-mails when patients arranged their consultation or contacted the clinic due to questions was established. In this e-mail patients were informed about the fact that the UDOC was open and that patients were seen regularly. Additionally, safety precautions with distancing in waiting areas, fever measurements, questionnaires and face masks when entering the clinic and a visiting ban were implemented. Since an essential drop in consultations has already been registered at the beginning of the pandemic, proactive cancellations and postponements of appointments in the PC by the clinic to respect COVID-19 preventive measures were not performed. Additionally, teledermatological consultations were offered, but was only enquired and carried out on one single patient.

## 5. Conclusions

It is extremely important to determine the impact and consequences of the SARS-CoV-2 pandemic on non-COVID-19-assoiciated diseases in order to prevent collateral damage. Particular patient groups—patients older than 85 years and patients with malignant, chronic inflammatory, and infectious diseases—were identified in this study to be more likely to be absent during the pandemic. Whereas some consultations can be postponed without far-reaching consequences, others—especially malignant diseases—must not be delayed in terms of correct diagnosis and prompt treatment. This might result into a potential backlog and more patients becoming lost to follow-up in the future. Identifying missed patients and catching up on their medical care apart from COVID-19 will pose an enormous challenge for health care systems globally in these days. Increased morbidity and especially mortality of non-COVID-19-related diseases in the future might be a consequence which urgently needs to be addressed and prevented.

Further wide-ranging interdisciplinary studies are in great demand to reveal neglected diagnoses due to SARS-CoV-2.

## Supplementary Materials

The following are available online at https://www.mdpi.com/1660-4601/17/17/6182/s1, Figure S1: CN per week and relative distribution of the UDOC. (A) Average CN per week between CW 2 and CW 9 from 2015 to 2019 compared to 2020. (B) Average CN per week between CW 10 and CW 16 from 2015 to 2019 compared to 2020. Beginning of the second accounting quarter in CW 14 (red arrow), first case of COVID-19 reported in Bavaria in CW 5 (blue arrow), declaration of state of emergency in Bavaria in CW 12 (green arrow). (C) Relative distribution of outpatient care regarding case numbers in March and April from 2015 to 2020. Subunits within the UDOC: Phototherapy (checkered), dermato-oncology (hatched), allergology (light gray), special consultation hours (middle gray), unselected outpatient clinic (polyclinic; black). CN, case number; CW, calendar week; UDOC, university dermatology outpatient clinic. Figure S2: Relative distribution of male (black) and female (gray) of actual and missed consultations and in the polyclinic in March and April. (A) Actual consultations in 2019 (*n* = 2609; male = 1311; female = 1298). (B) Missed consultations in 2019 (*n* = 370; male = 236; female = 134). (C) Actual consultations in 2020 (*n* = 1586; male = 875; female = 711). (D) Missed consultations in 2020 (*n* = 457; male = 271; female = 186). Figure S3: Average age in years of actual and missed consultations in the polyclinic between March and April. (A) Average age in 2019. (B) Average age in 2020.

## References

[1] Lu H.-Z., Stratton C.W., Tang Y.-W. (2020). Outbreak of pneumonia of unknown etiology in Wuhan, China: The mystery and the miracle. J. Med. Virol..

[2] Du Toit A. (2020). Outbreak of a novel coronavirus. Nat. Rev. Microbiol..

[3] Emanuel E.J., Persad G., Upshur R., Thome B., Parker M., Glickman A., Zhang C., Boyle C., Smith M., Phillips J.P. (2020). Fair Allocation of Scarce Medical Resources in the Time of Covid-19. N. Engl. J. Med..

[4] (2020). Corona-Pandemie/Bayern Ruft den Katastrophenfall aus/Veranstaltungsverbote und Betriebsuntersagungen. https://www.bayern.de/corona-pandemie-bayern-ruft-den-katastrophenfall-aus-veranstaltungsverbote-und-betriebsuntersagungen/.

[5] Wargon M., Guidet B., Hoang T.D., Hejblum G. (2009). A systematic review of models for forecasting the number of emergency department visits. Emerg. Med. J..

[6] Diehl A.K., Morris M.D., Mannis S.A. (1981). Use of Calendar and Weather Data to Predict Walk-In Attendance. South. Med. J..

[7] Holleman D.R., Bowling R.L., Gathy C. (1996). Predicting daily visits to a waik-in clinic and emergency department using calendar and weather data. J. Gen. Intern. Med..

[8] Batal H., Tench J., McMillan S., Adams J., Mehler P.S. (2001). Predicting patient visits to an urgent care clinic using calendar variables. Acad. Emerg. Med..

[9] Allocca M., Fiorino G., Furfaro F., Gilardi D., Radice S., D’Amico F., Zilli A., Danese S. (2020). Maintaining the Quality Standards of Care for Inflammatory Bowel Disease Patients During the COVID-19 Pandemic. Clin. Gastroenterol. Hepatol..

[10] Metzler B., Siostrzonek P., Binder R.K., Bauer A., Reinstadler S.J. (2020). Decline of acute coronary syndrome admissions in Austria since the outbreak of COVID-19: The pandemic response causes cardiac collateral damage. Eur. Heart J..

[11] Schwarz V., Mahfoud F., Lauder L., Reith W., Behnke S., Smola S., Rissland J., Pfuhl T., Scheller B., Böhm M. (2020). Decline of emergency admissions for cardiovascular and cerebrovascular events after the outbreak of COVID-19. Clin. Res. Cardiol..

[12] Garfin D.R., Silver R.C., Holman E.A. (2020). The novel coronavirus (COVID-2019) outbreak: Amplification of public health consequences by media exposure. Health Psychol..

[13] Zhou F., Yu T., Du R., Fan G., Liu Y., Liu Z., Xiang J., Wang Y., Song B., Gu X. (2020). Clinical course and risk factors for mortality of adult inpatients with COVID-19 in Wuhan, China: A retrospective cohort study. Lancet.

[14] Li X., Xu S., Yu M., Wang K., Tao Y., Zhou Y., Shi J., Zhou M., Wu B., Yang Z. (2020). Risk factors for severity and mortality in adult COVID-19 inpatients in Wuhan. J. Allergy Clin. Immunol..

[15] Bialek S., Boundy E., Bowen V., Chow N., Cohn A., Dowling N., Ellington S., Gierke R., Hall A., MacNeil J. (2020). Severe Outcomes Among Patients with Coronavirus Disease 2019 (COVID-19)—United States, February 12–March 16, 2020. MMWR. Morb. Mortal. Wkly. Rep..

[16] Wang H., Zhang L. (2020). Risk of COVID-19 for patients with cancer. Lancet Oncol..

[17] Xia Y., Jin R., Zhao J., Li W., Shen H. (2020). Risk of COVID-19 for patients with cancer. Lancet Oncol..

[18] Wu P.-H., Chuang Y.-S., Lin Y.-T. (2019). Does Herpes Zoster Increase the Risk of Stroke and Myocardial Infarction? A Comprehensive Review. J. Clin. Med..

[19] Johnson R.W., Rice A.S. (2014). Postherpetic neuralgia. N. Engl. J. Med..

[20] Cormier J.N., Voss R.K., Woods T.N., Cromwell K.D., Nelson K.C. (2015). Improving outcomes in patients with melanoma: Strategies to ensure an early diagnosis. Patient Relat. Outcome Meas..

[21] Pennie M.L., Soon S.L., Risser J.B., Veledar E., Culler S.D., Chen S.C. (2007). Melanoma outcomes for Medicare patients: Association of stage and survival with detection by a dermatologist vs a nondermatologist. Arch. Dermatol..

[22] Maringe C., Spicer J., Morris M., Purushotham A., Nolte E., Sullivan R., Rachet B., Aggarwal A. (2020). The impact of the COVID-19 pandemic on cancer deaths due to delays in diagnosis in England, UK: A national, population-based, modelling study. Lancet Oncol..

