# Supplementary material for: The Impact and Consequences of SARS-CoV-2 Pandemic on a Single University Dermatology Outpatient Clinic in Germany
Source: Int J Environ Res Public Health. 2020 Aug 26;17(17):6182. doi: 10.3390/ijerph17176182 (PMC7504311; doi:10.3390/ijerph17176182)
Supplement: Supplementary file 1 [file ijerph-17-06182-s001.pdf]

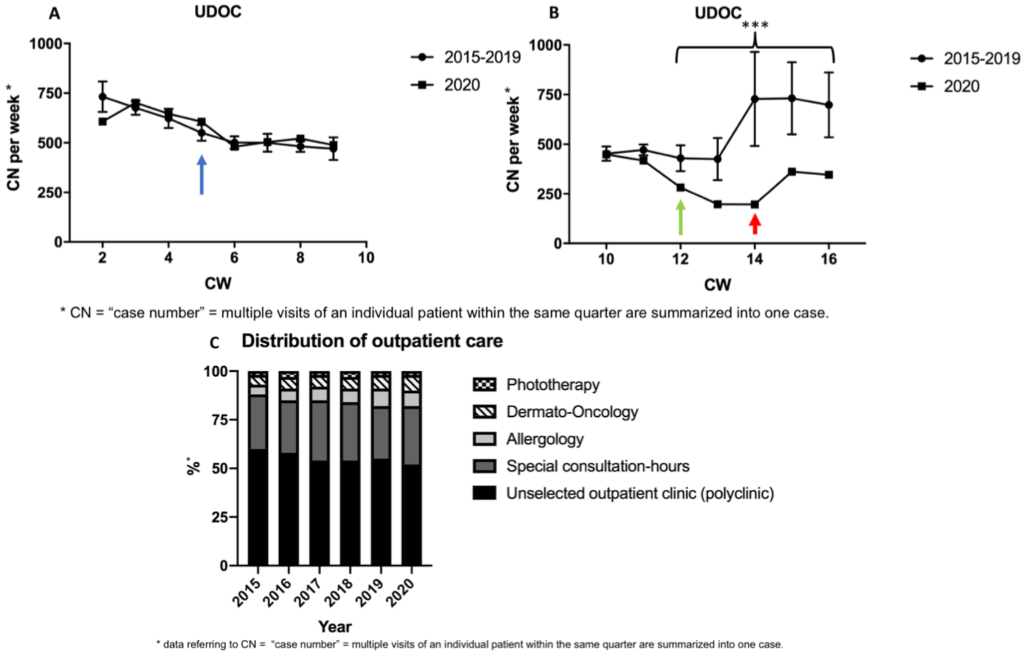

Figure S1. CN per week and relative distribution of the UDOC. (**A**) Average CN per week between CW 2 and CW 9 from 2015 to 2019 compared to 2020. (**B**) Average CN per week between CW 10 and CW 16 from 2015 to 2019 compared to 2020. Beginning of the second accounting quarter in CW 14 (red arrow), first case of COVID-19 reported in Bavaria in CW 5 (blue arrow), declaration of state of emergency in Bavaria in CW 12 (green arrow). (**C**) Relative distribution of outpatient care regarding case numbers in March and April from 2015 to 2020. Subunits within the UDOC: Phototherapy (checkered), dermato-oncology (hatched), allergology (light gray), special consultation hours (middle gray), unselected outpatient clinic (polyclinic; black). CN, case number; CW, calendar week; UDOC, university dermatology outpatient clinic.

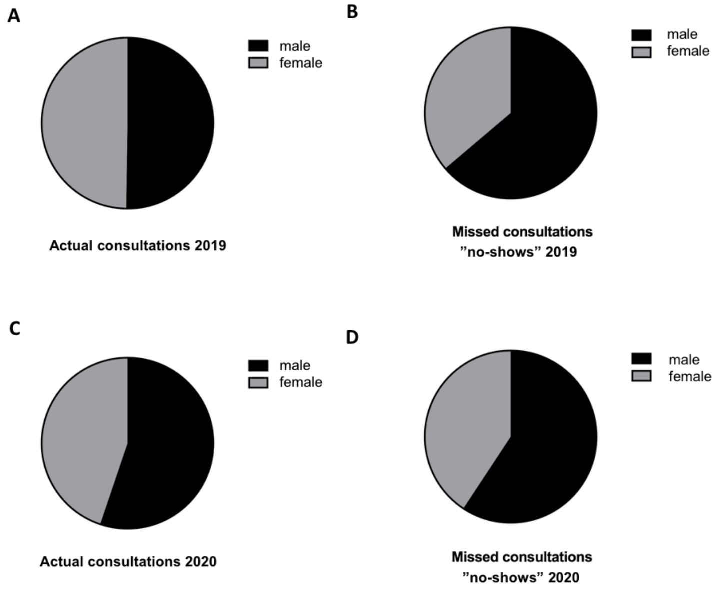

Figure S2. Relative distribution of male (black) and female (gray) of actual and missed consultations and in the polyclinic in March and April. (**A**) Actual consultations in 2019 (n = 2609; male = 1311; female = 1298). (**B**) Missed consultations in 2019 (n = 370; male = 236; female = 134). (**C**) Actual consultations in 2020 (n = 1586; male = 875; female = 711). (D) Missed consultations in 2020 (n = 457; male = 271; female = 186).

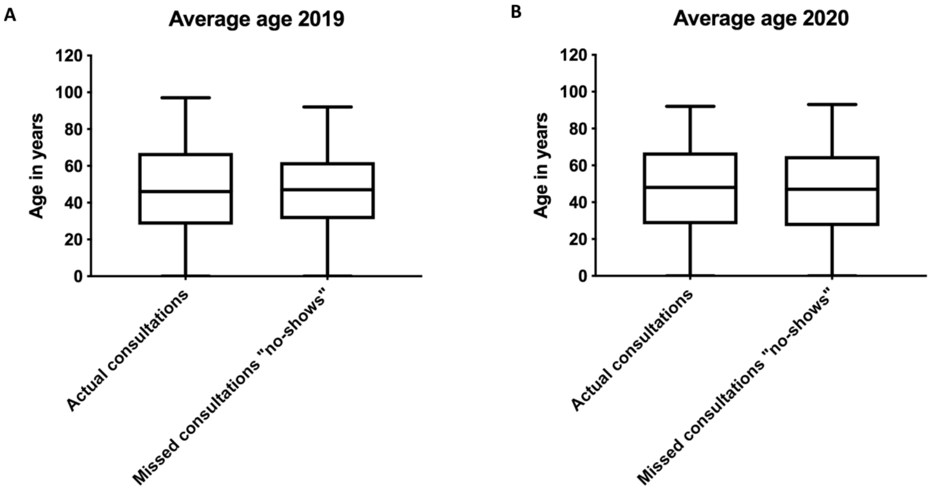

Figure S3. Average age in years of actual and missed consultations in the polyclinic between March and April. (**A**) Average age in 2019. (**B**) Average age in 2020.
